# Immunization against GAD Induces Antibody Binding to GAD-Independent Antigens and Brainstem GABAergic Neuronal Loss

**DOI:** 10.1371/journal.pone.0072921

**Published:** 2013-09-18

**Authors:** Thashi Chang, Harry Alexopoulos, Philippa Pettingill, Mary McMenamin, Robert Deacon, Ferenc Erdelyi, Gabor Szabó, Camilla J. Buckley, Angela Vincent

**Affiliations:** 1 Neuroimmunology Group, Weatherall Institute of Molecular Medicine and Nuffield Department of Clinical Neurosciences, John Radcliffe Hospital, University of Oxford, Oxford, United Kingdom; 2 Department of Physiology Anatomy and Genetics, University of Oxford, Oxford, United Kingdom; 3 Department of Experimental Psychology, University of Oxford, Oxford, United Kingdom; 4 Department of Gene Technology and Developmental Neurobiology, Institute of Experimental Medicine, Budapest, Hungary; 5 Department of Clinical Medicine, University of Colombo, Colombo, Sri Lanka; University of Wuerzburg, Germany

## Abstract

Stiff person syndrome (SPS) is a highly-disabling neurological disorder of the CNS characterized by progressive muscular rigidity and spasms. In approximately 60–80% of patients there are autoantibodies to glutamic acid decarboxylase (GAD), the enzyme that synthesizes *gamma*-amino butyric acid (GABA), the predominant inhibitory neurotransmitter of the CNS. Although GAD is intracellular, it is thought that autoimmunity to GAD65 may play a role in the development of SPS. To test this hypothesis, we immunized mice, that expressed enhanced green fluorescent protein (EGFP) under the GAD65 promoter, with either GAD65 (n = 13) or phosphate buffered saline (PBS) (n = 13). Immunization with GAD65 resulted in autoantibodies that immunoprecipitated GAD, bound to CNS tissue in a highly characteristic pattern, and surprisingly bound not only to GAD intracellularly but also to the surface of cerebellar neurons in culture. Moreover, immunization resulted in immunoglobulin diffusion into the brainstem, and a partial loss of GAD-EGFP expressing cells in the brainstem. Although immunization with GAD65 did not produce any behavioral abnormality in the mice, the induction of neuronal-surface antibodies and the trend towards loss of GABAergic neurons in the brainstem, supports a role for humoral autoimmunity in the pathogenesis of SPS and suggests that the mechanisms may involve spread to antigens expressed on the surface of these neurons.

## Introduction

Stiff person syndrome (SPS), first described by Moersch and Woltman in 1956, is a highly disabling, progressive disorder of the central nervous system (CNS) characterized by muscle rigidity and spasms [1], and recently reviewed by Duddy and Baker [2]. Autoantibodies to glutamic acid decarboxylase (GADAbs) have been reported in approximately 60 to 80% of patients with SPS [3], [4], [5]. GAD is the rate-limiting enzyme in the synthesis of the inhibitory neurotransmitter *gamma*-amino butyric acid (GABA), and impairment of GAD activity would be expected to lead to rigidity and spasms as a consequence of impaired motor inhibition. An autoimmune pathogenesis has been suggested in SPS because of its association with type-1 diabetes and GADAbs [6] and with other autoantibodies and autoimmune diseases [5], [7], a female preponderance, and the clinical improvement with immunotherapy. It has been suggested that autoimmunity to the GAD65 isoform of GAD may be one of the initiating events leading to the development of SPS [8].

One of the criteria for establishing the autoimmune nature of a disease is the induction of a disease model by immunization of experimental animals with the purified antigen [9]. Although autoimmune neurological diseases of the peripheral nervous system, such as myasthenia gravis, have been replicated in animal models by immunizing against the acetylcholine receptor [10], there have been relatively few reports of active immunization against CNS antigens. This may partly reflect resistance in believing that a CNS disease can be caused by autoantibodies due to the potential constraints on antibody entry into the CNS imposed by the blood brain barrier (BBB). However, when methods to overcome the BBB permeability have been successful, it has been possible to demonstrate central effects caused by peripherally induced autoantibodies [11]. Moreover, it is becoming increasingly clear that autoantibodies to CNS antigens are likely to cause disease in humans [12] with recent reports of successful induction of disease with intrathecal or intraventricular injection of human antibodies against the vesicular protein amphiphysin, or GAD65 [13] [14] [15]. However, to date, there have been no reports of attempts to develop SPS by active immunization against GAD65.

## Materials and Methods

### Mice

Transgenic mice on a background of CBA/C57Bl6 that express enhanced green fluorescent protein (EGFP) under the GAD65 promoter were used to monitor the number of GABAergic neurons visualized by EGFP expression [16]. Mice were housed in groups with water and food *ad libitum* in a non-specific pathogen free environment.

### Ethics Statement

All procedures were carried out in accordance with the UK Home Office guidelines under a project license granted by the Home Office to AV (Immunity in Neurological and Developmental Disorders, PPL No 30/1890). Compliance to rules and regulations and adherence to the 3Rs principles was monitored by Biomedical Services, University of Oxford and Home Office inspectors. Animals were sacrificed using an approved by the Home Office Schedule 1 procedure (exposure to carbon dioxide gas in a rising concentration).

### Experimental protocol

GAD-EGFP transgenic mice of 8 weeks of age were used. Because of limitations on the numbers, both females (n = 14, weight 18+/−1 g) and males (n = 1, weights 25.3+/−1 g) were immunized. The allocation to test or control was based on behavioral testing (see below). The mice were immunized subcutaneously with 20 µg of recombinant human GAD65 [(rhGAD65) kindly donated by RSR Ltd, Cardiff, UK] in PBS emulsified with complete Freund's adjuvant (CFA; Sigma-Aldrich, Poole, Dorset, UK) or with PBS in CFA (see Supplementary figure 1 for details). Three boosts were given at 28-day intervals with the same amount of rhGAD65 in incomplete Freund's adjuvant (IFA). Lipopolysaccharide (LPS) (Sigma-Aldrich) 3 mg/kg was injected intraperitoneally 10 days after the second boost and 3 days after the third boost to permeabilize the blood brain barrier [11].

### GADAb radioimmunoprecipitation and GADAb titration assays

Mouse sera were obtained at days 62, 97 and 155 (figure S1) and serially diluted in PT× (0.02 M phosphate, 0.1% Triton X-100, pH 7.2) at 1∶3 dilutions starting at 1 µl of serum; normal mouse serum was added to make up to a total of 2 µl of serum. 50 µl of freshly reconstituted ^125^I-labelled GAD65 (RSR Ltd) was added and incubated overnight at 4°C. The antigen-antibody complex was precipitated over 2 hours at room temperature with anti-mouse IgG (Binding Site Ltd, Birmingham, UK). The precipitate was centrifuged at 13000 rpm for 5 minutes, washed three times with PT× and radioactivity counted for 1 minute on a gamma counter (COBRA II, Auto-Gamma counting system, Packard, Meriden). The GADAb titer in U/ml for the sample was read from the standard curve and multiplied by the appropriate serum dilution factor.

### Behavioral analyses

Mice were allocated prior to immunization to balanced ‘Test’ or ‘Control’ groups by matching for their baseline burrowing performance, which is a species-typical behavior that is very sensitive to sickness and welfare problems. After immunization, they were observed for abnormalities in gait or other indication of stiffness or spasms. Behavioral tests included burrowing and the accelerating rotarod to evaluate general wellbeing and motor coordination; the light-dark box and the white open-field tests were used to evaluate the response to anxious and stressful environments [17], [18]. All behavioral tests were conducted at baseline and on four subsequent occasions over the immunization period by an investigator blind to the group allocation of the mice.

### Primary neuronal cultures

Primary cultures of cerebellar neurons were prepared from transgenic GAD65-EGFP P2 pups, as previously described [19]. Coverslips of cerebellar neurons at 7 days-in-culture were fixed in 4% paraformaldehyde (PFA) (Sigma-Aldrich) in PBS for 30 minutes at room temperature, washed and stored in PBS at 4°C until use. For non-permeabilized cells, the coverslips were rinsed with PBS (5 min×3) and non-specific binding blocked with 5% normal goat serum (NGS) (Vector Laboratories, Peterborough, UK) in PBS. The primary antibody was applied at a dilution of 1/200 in blocking solution and incubated at room temperature for 1 hour. Coverslips were rinsed with PBS (5 min×3) and fluorochrome conjugated secondary antibody (Alexa Fluor®568, Molecular Probes®, Life Technologies Ltd, Paisley, UK) was added at a dilution of 1/200 in 5% NGS in PBS and incubated at room temperature for 1 hour. Coverslips were then rinsed with PBS and mounted using fluorescent mounting medium (Dako, Glostrup, Denmark). Slides were allowed to air dry overnight at 4°C and visualized using confocal microscopy (BioRad Radiance 2000). For permeabilization, after fixation the coverslips were incubated in PBST (PBS with 0.3% Triton X-100) for 15 minutes at room temperature followed by further PBS rinses (5 min×2) and all further steps contained 0.1% Triton-X100.

To confirm the reactivity of the sera with intact neurons, live unfixed wild-type cerebellar cultures from P6 rat pups [20] were incubated with the test or control mouse sera 1/200 and detected with Alexa Fluor antibodies to the appropriate IgG species. The granule neurons were identified using an antibody to the GABA receptor alpha6 [21].

### Processing CNS tissue from mice

Mice deeply anaesthetized by halothane inhalation were transcardially perfused with chilled PBS (25 ml/mouse) followed by a freshly made filtered solution of 4% PFA (Sigma-Aldrich) in PBS (150 ml/mouse). Brains and spinal cords were removed and post-fixed in the same fixative for 30 minutes. All tissues were cryoprotected overnight at 4°C in a solution of 30% sucrose in Tris-buffered saline (TBS: 50 mM Tris, pH 7.6). Brains and spinal cords were sectioned into regions and mounted in Tissue-Tek® mounting medium (Sakura Finetek UK Ltd, Thatcham, UK) within cryomoulds on dry ice and stored at −80°C. Coronal and sagittal cryostat sections (12 µm-thick) were cut on chrome gelatin-coated slides, dried overnight in a vacuumed chamber, wrapped in foil and stored at −20°C until used for antibody detection. 40 µm-thick sections were collected in PBS, and stored at 4°C until use.

### Immunohistochemistry to detect mouse IgG in brain following active immunization

Endogenous peroxidases were inactivated by rinsing 40 µm-thick sections in TBS containing 1% H_2_O_2_ for 15 minutes. Following 3 rinses in TBS, the sections were incubated in 10% NGS (Vector Laboratories) in TBST (0.3% Triton X-100) for 2 hours to block non-specific binding. Sections were then incubated with biotinylated anti-mouse IgG (Vector BA-9200; 1∶500) in TBST containing 10% NGS overnight at 4°C with constant gentle agitation on a platform shaker. The next day, sections were rinsed in TBS (3×5 min) and incubated for 2 hours in Vectastain ABC (avidin-biotin) solution (Vector Laboratories). Sections were further rinsed in TBS (2×5 min, 1×30 min). Visualization was achieved by incubating sections in 0.05% DAB (Sigma-Aldrich) in TBS+6 µl H_2_O_2_ for 5–8 min protected from light. The reaction was terminated by transferring sections into cold TBS followed by further rinses in TBS (3×5 min). Sections were mounted on chrome gelatine coated slides and air-dried overnight at room temperature. Coverslips were applied over a xylene based mounting solution (Histomount, RA LAMB, HS 103) and viewed under a light microscope (Nikon Eclipse E400). For identification of mouse GAD, a mouse monoclonal antibody that binds both human and mouse GAD65/67 was used (Stressgen, Canada).

### Immunostaining of brain sections for neuronal counting

Immunostaining was carried out using the Shandon Sequenza slide racks with coverplate system (Fisher Scientific Ltd, Loughborough, UK). 12 µm-thick sections were washed with two runs of PBS. Non-specific binding was blocked with 10% NGS in 0.3% PBST at room temperature for 1 hour. Primary antibodies were applied for one hour in 10% NGS in 0.3% PBST. After 3 washes in PBS, the secondary antibodies were applied for 1 hour. Slides were washed with PBS (3×5 min) and coverslips applied on fluorescent mounting medium (Dako). Slides were allowed to dry at 4°C overnight before examination under confocal microscopy.

GABAergic (GAD) neurons were identified by the green fluorescence emitted by the EGFP co-expression, and the neuron-specific mouse anti-NeuN antibody (1∶1000; Chemicon), or rabbit anti-calbindin antibody (1∶1000; Swant, Switzerland) for the cerebellum, were used to identify the total number of neurons. Five regions each in the cerebellum, hippocampus, brainstem, cervical, thoracic and lumbar cords were selected and labeled 1 to 5 and the tissue sections from each region photographed using the confocal microscope. Neurons were counted in the frame only if part or the entire nucleus was within the frame. To reproduce the results, further three mice were immunized with GAD65 or PBS and the analysis was repeated using 12 sections from each mouse brain starting from the midline and progressing laterally whilst collecting every third section and discarding two. In both experiments, the micrographs were analyzed using ImageJ cell counter software 1.36b (National Institutes of Health, USA). Neuronal counting was performed by two independent investigators, blinded to mice group allocation and the mean counts were used in the analysis.

### Immunohistological analysis of brain sections for an inflammatory response, synaptic loss or apoptosis

Immunostaining of brain sections was performed as described above for neuronal counting. Hamster anti-CD3 (1∶50; Caltag laboratories, Burlingame, CA94010) (T cells), rat anti-CD45 (1∶50; Caltag laboratories, Burlingame, CA94010) (human leucocyte antigen), rat anti-F4/80 (1∶100; Serotec) (microglia), rabbit anti-GFAP (1∶1000; Dako) (astrocytes), and anti-mouse CD16/CD32 (FcγIII/II receptor) (1∶50; BD Pharmingen) were applied as primary antibodies to detect an inflammatory response in immunized mouse brain tissue. Rabbit anti-synaptophysin (1∶100; NeoMarkers, Fremont, CA) were used to detect any loss of synapses whilst rabbit anti-AC3 (1∶200; Cell Signalling Technology) and TUNEL test (Roche, UK) were used to detect apoptosis. Secondary antibodies were specifically directed against the source of species of the primary antibodies and conjugated with fluorophores with excitation maxima compatible with the confocal microscope lasers. All secondary antibodies were from Alexa Fluor®568, Molecular Probes®, Life Technologies Ltd.

### Statistics

Two-way ANOVAs were used for the behavioral tests to compare test and control results. For the neuronal counts, the test and control results in different brain and spinal cord regions were compared with unpaired t tests.

## Results

### Antibody response in mice actively immunized with rhGAD65

**Figure 1:**
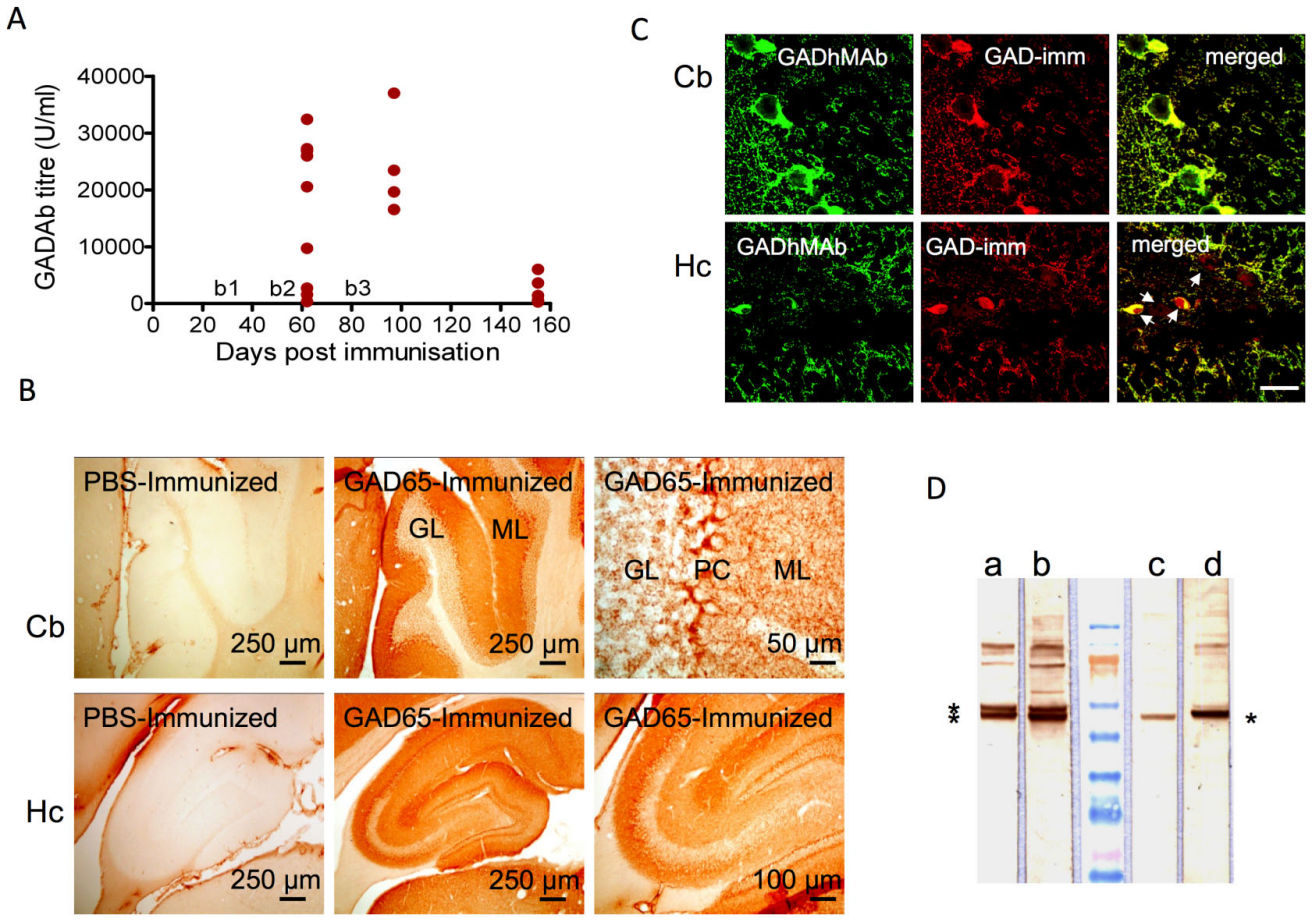
GADAb titers and immunoreactivity in mice immunized with recombinant human GAD65. A. Three boosts (b1, 2, 3) were given at 4-week intervals after the initial immunization (D0). Results were analyzed at 10 days after the second boost (D62, n = 9), 15 days (D97, n = 4) and 73 days (D155, n = 5) after the third boost. All mice immunized with PBS in the control group had a GADAb titer of <1 U/ml at all three time points. B. Immunoreactivity of sera from mice immunized with PBS and GAD65 on rat brain sections from the cerebellum (Cb) and hippocampus (Hc). Sera from GAD65-immunized mice showed immunoreactivity typical of GADAbs. In contrast, sera from PBS-immunized mice did not show any immunoreactivity. GL: granular layer; PC: Purkinje cell layer; ML: molecular layer. C. Confocal images of GADAb-positive mouse serum (GADAb titer 37000 U/ml) and monoclonal human GADAbs (GADMAb, a commercial antibody binding both human and mouse GAD65 and GAD67) on rat brain sections of cerebellum (Cb) and dentate gyrus of the hippocampus (Hc). The mouse serum antibodies colocalized with the monoclonal antibodies, although some additional reactivity of the monoclonal, appeared in the hippocampus (arrows). Scale bar = 25 µm. D. Two high titer mouse sera (c,d) shown binding to a band on Western blots of mouse cerebellum, similar to the lower of the two bands identified with the anti-GAD65/67 monoclonal antibody (a 1∶200 , b 1∶100).

The GAD65-immunized mice had high GADAbs titers by day 10 after the second boost (figure 1A), ranging from 350 to 32428 U/ml (mean 16386; median 20547) with only one serum having less than 1000 U/ml. The GADAb titers subsequently increased further (four mice; mean 24161 U/ml) but fell substantially by the time the experiment ended 73 days after the third boost (five mice; mean 2385), although even at this time, 3 of 5 mice still had titers more than 1000 U/ml. None of the mice immunized with PBS alone had detectable GADAbs at any of the three time points examined (not shown). None of the GAD65-immunized mice had glucose in their urine on analysis with reagent strips (data not shown), which excluded diabetes mellitus.

On immunohistochemistry, sera from GAD65-immunized mice demonstrated immunoreactivity on permeabilized rat brain sections that was not seen in the PBS-immunized mice (shown for cerebellum and hippocampus in figure 1B). The GAD65 positive binding was very similar to that of a monoclonal antibody to GAD (figure S2), and co-localized with the monoclonal antibody by immunofluorescence (figure 1C). Moreover, the high titer mouse sera bound to a band on western blots of mouse cerebellum homogenate, equivalent to the smaller of the two bands (kD65 and 67) identified by the monoclonal antibody.

**Figure 2:**
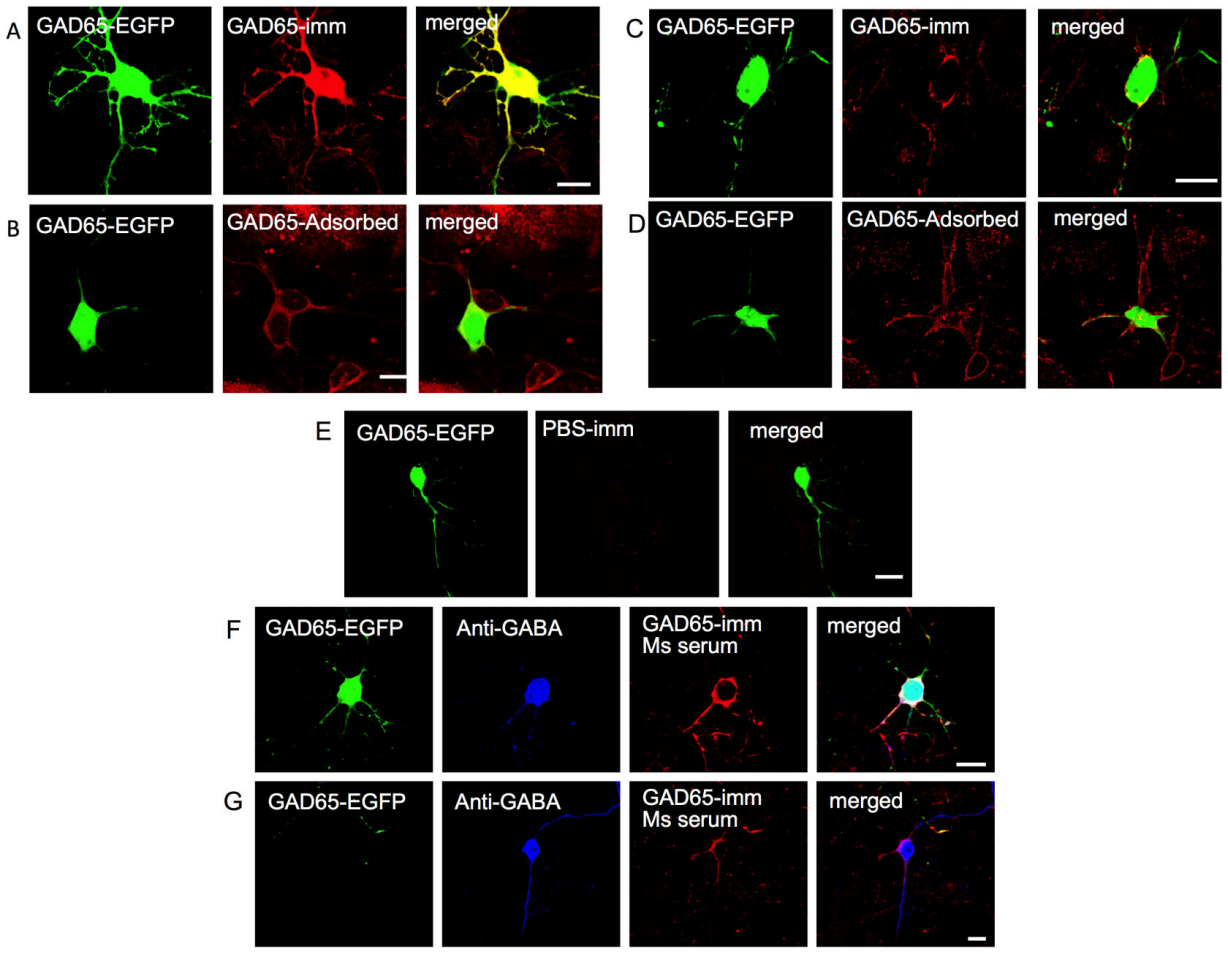
Confocal images of GADAb-positive mouse serum binding to cerebellar neurons in primary culture derived from GAD65-EGFP transgenic mice. A. Mouse serum antibodies bind intracellularly to permeabilized neurons. B. After absorption against GAD65, the same serum binds the surface of permeabilized neurons. C. Mouse serum antibodies bind to the surface of unpermeabilized neurons. D. Binding to unpermeabilized neurons is not reduced following adsorption against GAD65. E. PBS-immunized mouse serum did not show any specific binding to permeabilized or unpermeabilized neurons. F, G. GAD-Ab positive serum binds to the surface of EGFP-expressing, non-permeabilized neurons and few non-EGFP neurons. All neurons that showed surface binding were GABA positive. Scale bar = 10 µm. H. Non-confocal images of live, unfixed, rat cerebellar cultures co-stained with MAP-2 (red) and antibody to GABA receptor alpha6 (GABARA6; H, left panel, green) or with MAP-2 (red) and PBS- or GAD65-immunized mouse sera showing binding to the surface of some neurons with a punctuate pattern (green, I, J).

Sera from GAD65-immunized mice and controls were applied to primary neuronal cultures derived from the GAD65-EGFP transgenic mice. When the serum was applied to permeabilized cultures, strong intracellular binding was seen on all EGFP-expressing neurons (figure 2A), as expected for neurons that express GAD65. Pre-incubation with recombinant-human (rh) GAD65 prevented the intracellular binding, confirming that it was directed against GAD65, but surprisingly revealed that there was still IgG binding to the surface of the EGFP-GAD65-expressing neurons (figure 2B). Moreover, when applied to unpermeabilized cultures, mouse IgG bound to the surface of all EGFP-expressing neurons and also to some non-EGFP expressing neurons (figure 2C); this was not unexpected since not all GAD neurons express EGFP. These neuronal surface binding antibodies were not adsorbed by pre-incubation with rhGAD65 (figure 2D). PBS-immunized mouse sera did not show any immunoreactivity with either permeabilized or unpermeabilized neurons in primary cerebellar cultures (eg. figure 2E). The non-EGFP neurons to which mouse IgG bound were GABAergic as shown by co-localization with commercial antibodies to GABA (figure 2F, 2G).

To confirm that the antibodies could bind to the surface of some cerebellar neurons, and that this was not confounded by the expression of the EGFP-GAD transgene, we also tested binding to rat cerebellar neurons, identified by their wide expression of GABAA6 receptors (figure 2H) or MAP-2. The mouse GAD-immunized sera bound to the surface of individual live neurons in these cultures (figure 2I) in a punctuate manner (figure 2J).

#### Behavioral analyses

**Figure 3:**
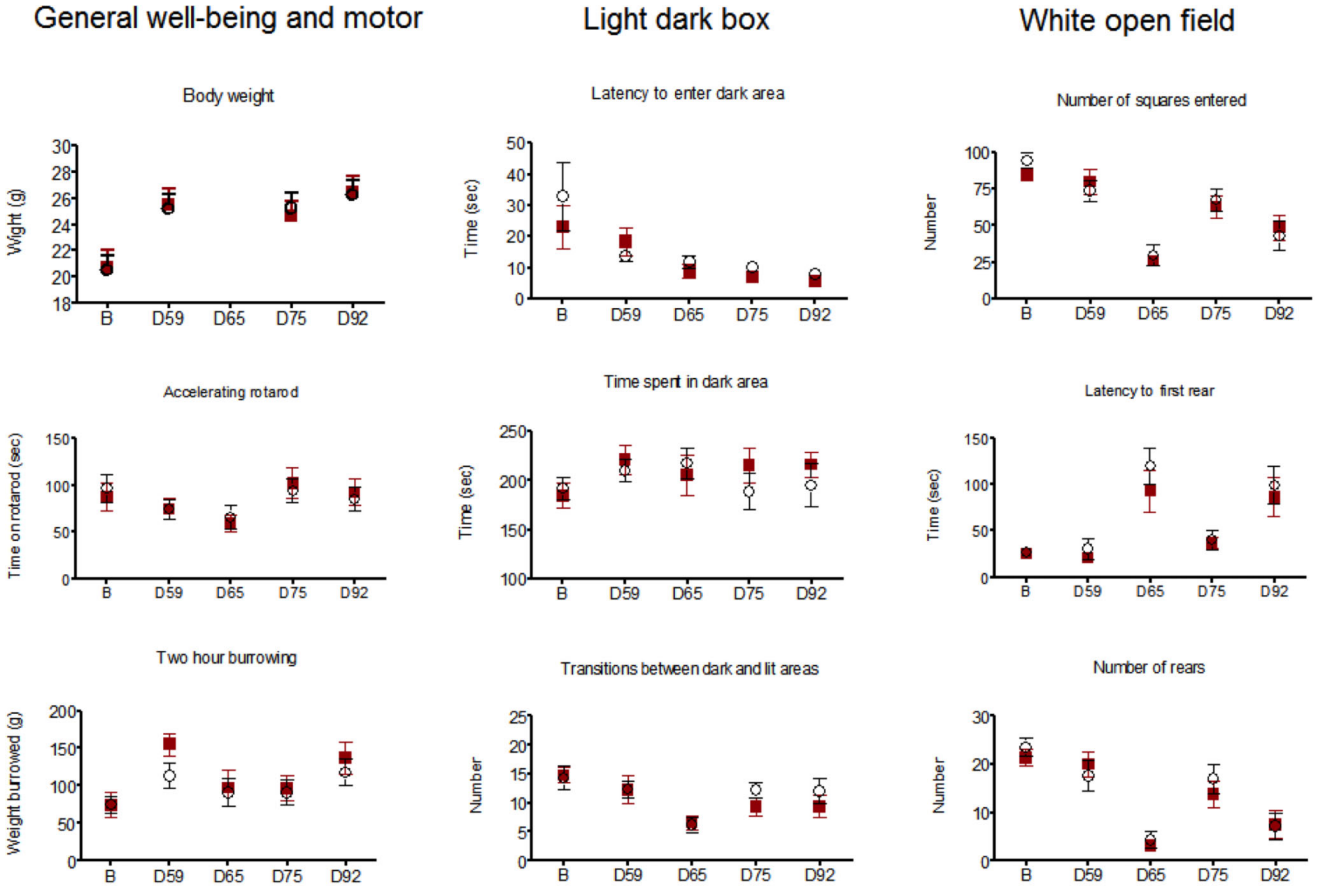
Behavioral analyses on GAD-immunized (n = 9; 6F, 3M) or PBS-immunized (n = 12; 8F, 4M) mice. Boosts were performed at days (D) D28, D52 and D82, and LPS given at D62 or D64 and D85. The results are given at baseline (B) and the different time points after the first immunization: D59, 1 week after the second boost; D65, 1 day and D75, 11 or 13 days after first LPS; and D92, 1 week after second LPS following third boost. The means and SEMs are shown at each time point for rhGAD65 (▪) and PBS (**○**) immunized mice. Although both females and males are included, neither these results nor analysis of the females only (6 Test and 8 Controls) revealed any differences between the two groups at any of the time points (2-way ANOVA).

Motor coordination (accelerating rotarod), general well-being (burrowing) or anxiety (white open-field and light-dark box) tests were performed at baseline and four later stages during the immunization protocol on coded mice. When tested at day 65, one day after LPS injection, some aspects of exploratory activity were altered in both test and control mice, particularly in the white open field. This was not seen when exploratory activity was tested at day 92, 7 days after LPS. There were no differences between control and test groups at any time point (Two-way ANOVA; figure 3).

#### Immunohistochemical analysis of IgG and T cell infiltrates in mouse brain following immunization with rhGAD65

**Figure 4:**
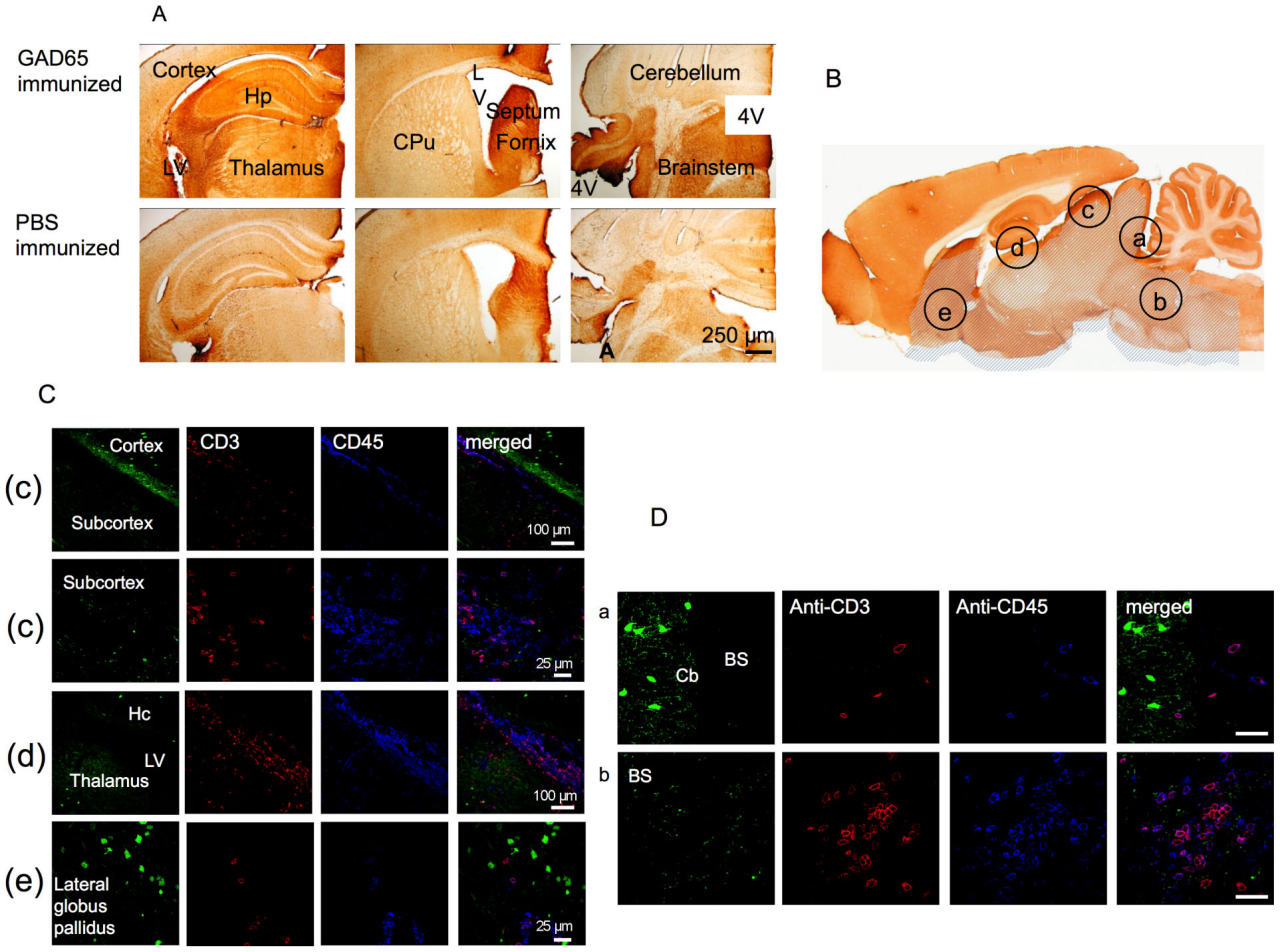
Staining for mouse IgG and T cell infiltrates in mouse brain following immunization with rhGAD65 or PBS. A. Coronal sections through hippocampus (Hp) and thalamus, striatum (CPu) and fourth ventricle (4V) were stained with anti-mouse IgG antibodies. Note the strong immune reaction (brown deposit) in the hippocampus, thalamus, septum, brainstem, adjacent to the ventricles and the lateral access of the fourth ventricle. Intensity of immunoreactivity appeared greater in GAD65 immunized mice brains compared to PBS immunized mice brains but this was not quantified further. LV = lateral ventricle. B. Sagittal section of a rodent brain showing the five regions investigated for cellular immunopathology. C. Confocal images showing T cells (CD3, red filter) and leucocytes (CD45, blue filter) in the brain regions. Some cells were identified in the subcortex, in the thalamus, the lateral ventricles and the lateral globus pallidus, but there were very scarce infiltrates in the cortex and hippocampus. D. Higher power images through the cerebellum and brainstem of the one GAD-immunized mouse with T cell (CD3, red filter) and leucocyte (CD45, blue filter) infiltrates in the brainstem, which were not found in the cerebellum. Note that the brainstem but not cerebellum was positive for IgG deposits (A). BS = brainstem; Cb = cerebellum. Scale bar = 25 µm.

In order to evaluate how much of the circulating IgG in mice had gained access to the CNS across the BBB, brains from the immunized mice were immunostained for mouse IgG. Anti-mouse IgG binding was surprisingly common in some brain areas, although not in the cerebellum and, although clearly present in both test and control, it appeared more extensive and intense in GAD65-immunized mice than in PBS-immunized mice (figure 4A); this difference was most apparent in the regions of the hippocampus, thalamus, brainstem and cortex but was not quantified. The immunohistochemical binding pattern did not demonstrate the characteristic immunohistochemical pattern described for GAD-Abs (figure 1B, figure S2), and thus the mouse IgG did not appear to have bound specifically to the GAD-expressing GABAergic neurons.

Brains and spinal cords from five mice from the test group and four mice from the control group were analyzed for inflammatory infiltrates, synaptic loss and apoptotic neurons in five different brain regions (figure 4B). Cellular infiltrates, both CD45 (leukocyte) and CD3 (T cell) positive, were found in the lining of the ventricles but there were no infiltrates detected in the cortex, hippocampus, cerebellum and beyond the caudal third of the brainstem, or in the spinal cord (figure 4C and data not shown). Only one mouse of the test group showed a heavy infiltrate of CD3 and CD45-positive cells in the subcortical and brainstem regions (figure 4C,D).

Astrocyte activation and expression of Fc receptors were not apparently different between the test and control brains; microglial activation was evident in the linings of ventricles and meninges but none was identified in the brain parenchyma. There was no apparent loss of synaptophysin stain or increase in apoptosis in the test mouse brains or spinal cords over and above that of the controls and these results were was not analyzed further.

### Neuronal loss in immunized mouse brains

**Figure 5:**
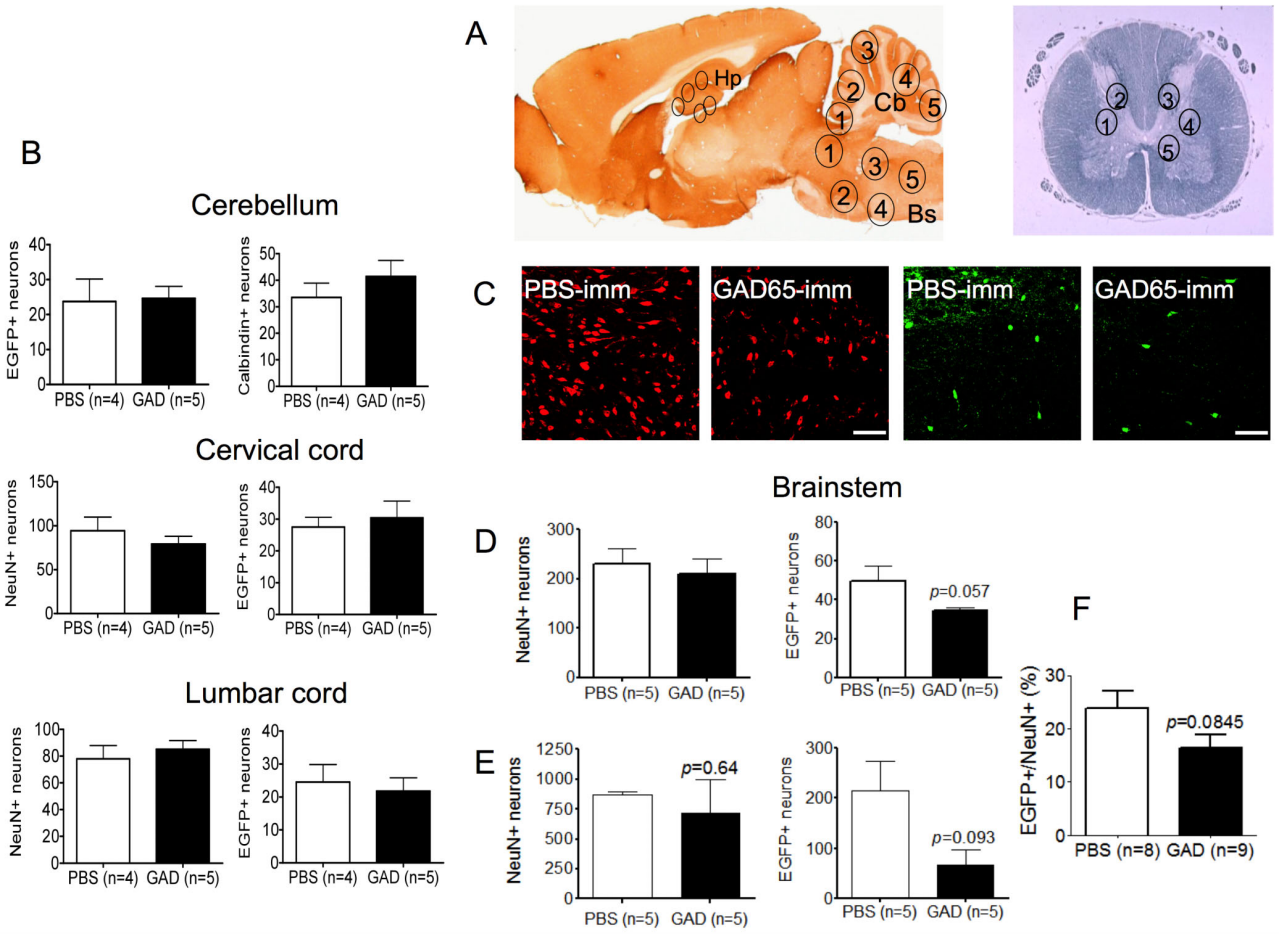
Neuronal counts and analysis following immunization A. Regions selected for neuronal counting (numbered 1 to 5), in the hippocampus (Hp), cerebellum (Cb) and brainstem (BS) are shown on a sagittal rodent brain section and in the grey matter on a coronal section of the spinal cord. B. EGFP-positive and total neuronal counts (calbindin+) in cerebellum, and (NeuN+) in two of the three spinal cord regions sampled. No differences were found between test and control mice. C. Representative images from region 5 of the brainstem demonstrating reduced numbers of EGFP+ neurons in the GAD65-immunized mice, compared with PBS-immunized controls, with some apparent reduction in the NeuN+ neurons. D. Counts obtained from the blinded analysis of all five brain stem regions. Although there was no significant reduction in total neurons, the EGFP+ cells showed a trend towards lower numbers in the brainstem (unpaired t test). E. To investigate further, the immunization and neuronal counting was repeated on three further GAD65 and PBS immunized mice, analyzing a larger number of neurons in the same regions of the brainstem with similar results. All neuronal counting was performed on coded sections by an independent investigator. Scale bar = 100 µm. F. To normalize between the results of D. and E., the EGFP-positive neurons were expressed as a percentage of the NeuN positive neurons for each slice.

To look for loss of GAD expressing neurons in the CNS, firstly NeuN antibody-positive neurons (or calbindin-positive neurons in the cerebellum) and EGFP-positive neurons were counted in regions of the hippocampus, cerebellum, brainstem and spinal cord (figure 5A). The EGFP positive cells in the hippocampus were consistently very few in number (mean 4/mouse) and were not analyzed further. Neither the total neurons nor the GAD-EGFP-positive neurons were different between test and control brains in the cerebellum or three spinal cord regions (two shown in Figure 5B). By contrast, the number of GAD-EGFP expressing neurons in the brainstems of GAD-immunized mice was lower although not reaching significance (*p* = 0.057; figure 5D). To confirm this potentially important result, we repeated the immunization, analyzing higher numbers of EGFP-positive neurons for comparison between three mice in each group. Again, there was a trend towards a reduction in the GAD-EGFP expressing neurons (*p* = 0.093, figure 5E). This trend was confirmed when the results of all experiments were normalized by expressing the EGFP positive cells as a percentage of the total NeuN positive neurons (figure 5F).

## Discussion

The presence of GADAbs in patients with stiff person syndrome has been known for almost three decades but because GAD is an intracellular enzyme the pathogenic significance of these antibodies has been in doubt. Our data demonstrate two potentially important findings. Firstly, immunization against GAD, which induced very high GADAb levels similar to those found in patients with SPS, resulted in IgG reactivity not only against the intracellular compartment of the GAD-EGFP expressing neurons, where GAD65 is located, but also against the extracellular surface of unpermeabilized GAD-EGFP positive and negative cerebellar neurons in both mouse and rat cultures. Secondly, in two independent and coded experiments, there was a trend towards reduced numbers of GAD-expressing neurons in the brainstem of the immunized mice. The apparent loss of GAD-EGFP expressing neurons suggests that antibodies, either against GAD or against an extracellular epitope, or possibly GAD-specific T cells that would also have been induced by the immunizations, are involved in the pathogenesis of SPS.

The antibody responses to immunization resulted in GADAb titers in mice that were comparable to or even higher than titers detected in SPS in humans. The high GADAb titers were sustained over a period of 5 months by boosts to allow a prolonged exposure of the mouse CNS to the immune response. The additional presence in the mouse sera of autoantibodies to surface epitopes of GABAergic neurons was a novel finding and interestingly similar reactivity has been observed in sera of SPS patients [22]. Since the intracellular antibody reactivity was adsorbed by rhGAD65, these findings suggest that either there is a distinct GAD epitope expressed on the surface of the neurons, antigenically different from GAD, or that the immunization results in some form of antigenic intermolecular spreading to other GABAergic neuronal antigens. One possibility would be that the mouse antibodies bound to GAD67 on the neuronal surface, but GAD67 is also considered an entirely intracellular antigen and western blotting in Figure 1 indicated low reactivity with GAD67. Another possibility is that there was antigen spreading. Both intra- [23] and inter-molecular spreading [24] have been identified in other experimental models, but the timing of the phenomena that we observed and how they relate to the GAD65 antibody response has not yet been explored. Western blotting did not give any evidence of additional bands (which one might expect in antigen spreading), but neuronal surface antibodies are usually directed to membrane antigens, which infrequently retain their antigenicity on western blots. Each of these aspects requires further study.

To try to maximize any potential effects, we used LPS to “permeabilize” the BBB. LPS has been recognized as a tool for this purpose for some time and was successful employed in a mouse model employing immunization against an epitope of the NMDA receptor [11]. However, care needs to be taken with the timing of its administration, as we found that some mouse behaviors were altered when tested the day following its administration. Our immunohistological analysis confirmed the presence of mouse IgG in some regions of the CNS, in both test and control animals, which may partly result from the LPS administration. The IgG staining appeared greater in the GAD-immunized mice, and was similar to that reported by Hansen et al 2013 [14] and seen by us in human SPS antibody mice injected systemically [22]. The finding of IgG in the brainstem, among other regions, suggests that this region is vulnerable to antibody-mediated conditions, and is consistent with the trend towards a reduction in EGFP+ neurons in the brainstem in mice immunized with GAD65 that we found both in the main experiment (p = 0.057) and in a repeat immunization (p = 0.093). EGFP is not expressed in all GABAergic neurons in these GAD-EGFP transgenic mice, and it would have been better to directly quantify GAD65 neurons; however, because GAD and GABA are very widespread in the neuropil as well as the cell bodies of inhibitory neurons, we found it difficult to delineate the cells for analysis with image J. Future experiments should measure GABA or GAD directly in tissue extracts.

GABAergic neurons arising from the lower medial brainstem (‘the inhibitory centre of Magoun’) and inhibitory interneurons within the medullary reticular formation have been shown to modulate axial muscle tone [25], [26], [27]. Hence, a loss of GABAergic inhibitory neurons in the brainstem could explain an increased excitability of spinal motor neurons that would result in axial rigidity and muscle spasms as seen in SPS. Moreover, mutant *hyrt* mice with lower levels of GABA_A_ receptors in the brainstem and spinal cord, exhibited stiff gait, hunched posture and stimulus-sensitive jerky movements, and on EMG demonstrated continuous motor unit activity (CMUA) at rest, reminiscent of SPS [28]. Therefore, even in the absence of an overt clinical or behavioral phenotype, electrophysiological analysis of brain slices from immunized mice could be performed in future to detect the presence of subclinical defects in inhibitory neuronal activity.

Our results do not exclude a role for GAD65-specific T cells which one would expect to have been induced initially and may have peaked earlier during the immunization, before we were able to investigate the brain pathology. One of our mice immunized against GAD65 demonstrated an inflammatory cell infiltrate that was conspicuously confined to subcortical and brainstem regions. It is likely that this mouse either had a more florid inflammatory response, or a slower clearance of the cellular infiltrate from the CNS, or both. Other evidence supports the contribution of T cells. Mice expressing a monoclonal GAD65-specific CD4+ T cell population developed a lethal encephalomyelitis-like disease in the absence of any other T cells or B cells, although they did not exhibit an SPS phenotype [29], and recently T cell infiltrates have been identified in the temporal lobes of three GAD-antibody positive patients with limbic encephalitis [30], but these observations do not exclude a role for autoantibodies to the neuronal surface as demonstrated here.

The clinical diversity of SPS includes variants such as stiff limb syndrome, progressive encephalomyelitis with rigidity and myoclonus, jerking SPS and paraneoplastic SPS. In addition, GAD antibodies at equally high titers have been found in patients with cerebellar [31] and epileptic [32] syndromes. It is not clear how this diversity of clinical features occurs in humans, and our attempts to induce disease by systemic injection of SPS IgG into mice were not successful [22], but some aspects of SPS have recently been modeled successfully in rats; injection of purified SPS IgG intrathecally produced anxiety-like behaviors [15] and intraventricular injections of IgG from a different patient produced motor dysfunction [14]. Moreover, as mentioned above, there were deposits of IgG in some regions of the brain [14]. The absence of clinical manifestations in our mice may reflect the fact that the GABAergic neuronal loss did not reach the threshold required to manifest axial rigidity and spasms.

We conclude that although immunization with GAD65 did not produce an overt clinical phenotype of SPS in mice and the loss of GABAergic neurons in the brainstem did not quite reach statistical significance, nevertheless these findings suggest an integral role for GAD65 immunity in the pathogenesis of SPS but a role which includes the development of novel cell-surface antibodies which might perhaps begin to provide a basis for the phenotypic diversity. Further studies are required to explore in more detail the roles of GAD-specific antibodies, the targets of the novel neuronal surface binding antibodies that we identified in this model, and in patients with GAD antibodies [22], and their effects on GABAergic neuronal functions.

## Supporting Information

Figure S1 Active immunization (A) Study profile and (B) time line for active immunization of GAD65-EGFP mice with rhGAD65. Note that 4 mice from the test group and 1 mouse from the control group had to be sacrificed because of ulcerating granulomas following immunization. tg = transgenic; BT = behavioral tests; LPS = lipopolysaccharide; SC = subcutaneous; CFA and IFA = complete and incomplete Freund's adjuvant; D = day.
(TIF)

Figure S2 **GAD immunoreactivity in rat brain sections.** (A) Whole rat brain saggital sections stained with healthy control and GAD monoclonal antibodies (×10). Note the contrast in immunoreactivity. (B) Examples of immunoreactivity in saggital sections through cortex, basal ganglia, hippocampus and cerebellum. First column represents normal healthy serum. Second and third columns represent GADmAb. Note (c) immunoreactive dots outlining pyramidal cells in the cortex (arrow heads); (e) intense immunoreactivity of lateral globus pallidus (LGP) relative to the striatum and thalamus (Th) in basal ganglia; (h, i) staining surrounding pyramidal cells in the hippocampal regions CA1-3; (l) the dense accumulation of immunoreactivity at the axon hillock (small arrows) and puncta that outline the perikaryon and dendritic tree of Purkinje cells (PC), punctate staining in the molecular (ML) and granular layer, and (k) intense immunoreactivity in deep cerebellar nuclei (DCn). SO: stratum oriens; SR: stratum radiatum; DG: dentate gyrus.
(TIF)
