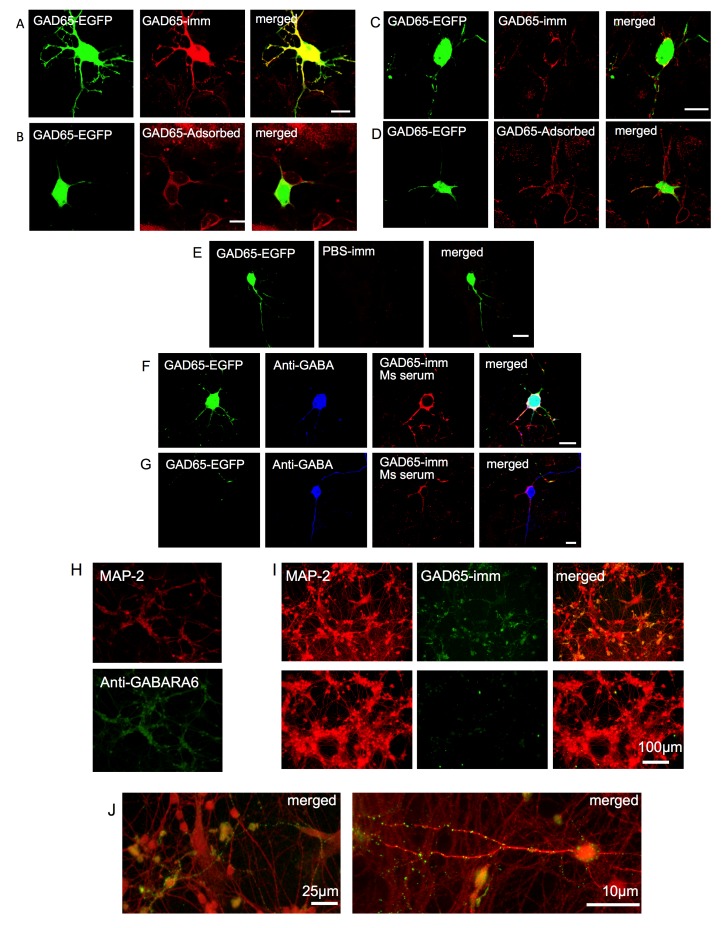# Correction: Immunization against GAD Induces Antibody Binding to GAD-Independent Antigens and Brainstem GABAergic Neuronal Loss

**DOI:** 10.1371/annotation/e71a42ed-eebf-43c1-b2f5-6ea70e9d8966

**Published:** 2013-10-25

**Authors:** Thashi Chang, Harry Alexopoulos, Philippa Pettingill, Mary McMenamin, Robert Deacon, Ferenc Erdelyi, Gabor Szabó, Camilla J. Buckley, Angela Vincent

The version of Figure 2 that appears in the article was incomplete. The correct version is available here: 

**Figure:**