# Supplementary figures and images for: Immunization against GAD Induces Antibody Binding to GAD-Independent Antigens and Brainstem GABAergic Neuronal Loss
Source: PLoS One. 2013 Sep 18;8(9):e72921. doi: 10.1371/journal.pone.0072921 (PMC3776810; doi:10.1371/journal.pone.0072921)

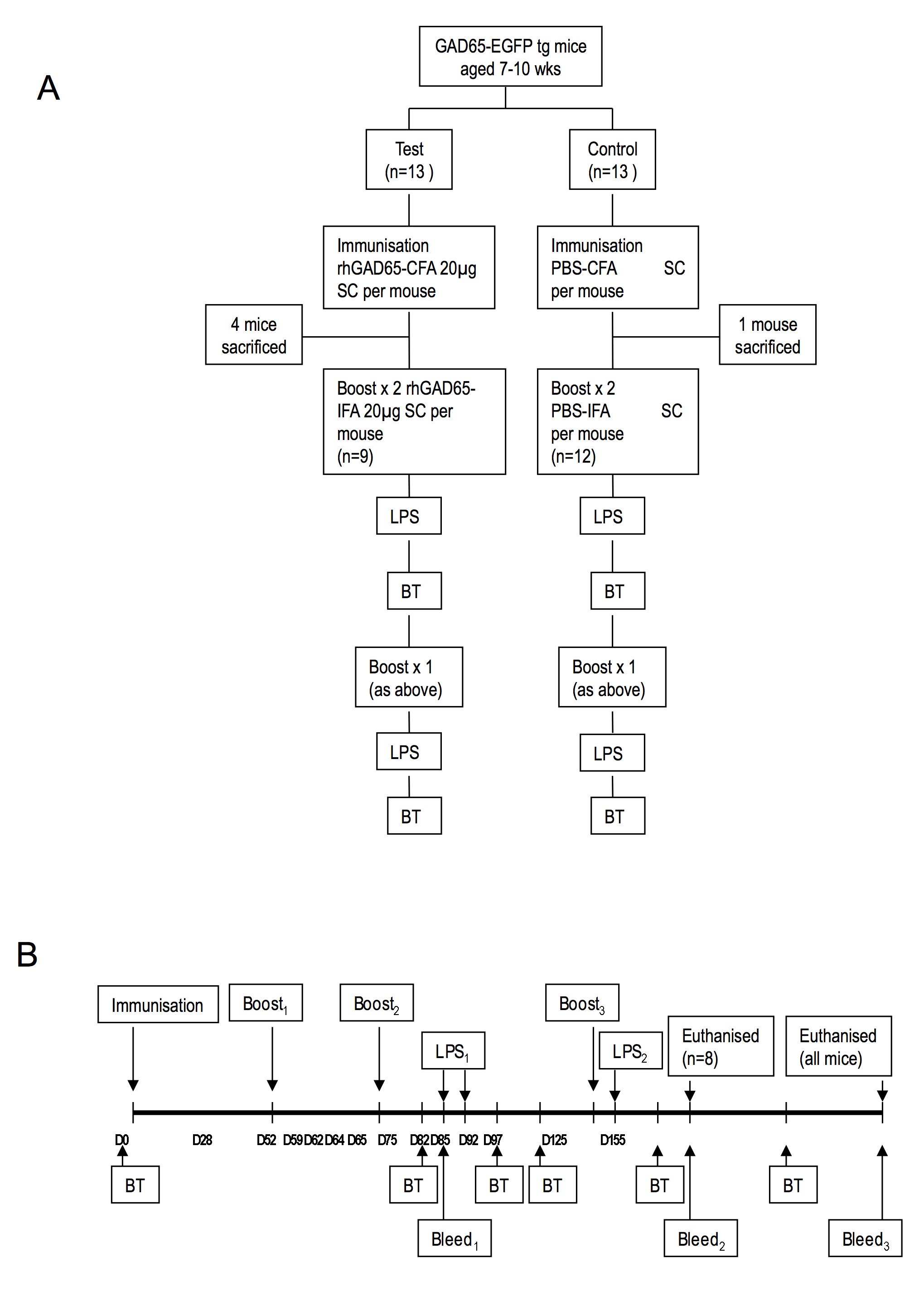

Supplement: Figure S1 — Active immunization (A) Study profile and (B) time line for active immunization of GAD65-EGFP mice with rhGAD65. Note that 4 mice from the test group and 1 mouse from the control group had to be sacrificed because of ulcerating granulomas following immunization. tg = transgenic; BT = behavioral tests; LPS = lipopolysaccharide; SC = subcutaneous; CFA and IFA = complete and incomplete Freund's adjuvant; D = day. (TIF) [file pone.0072921.s001.tif]

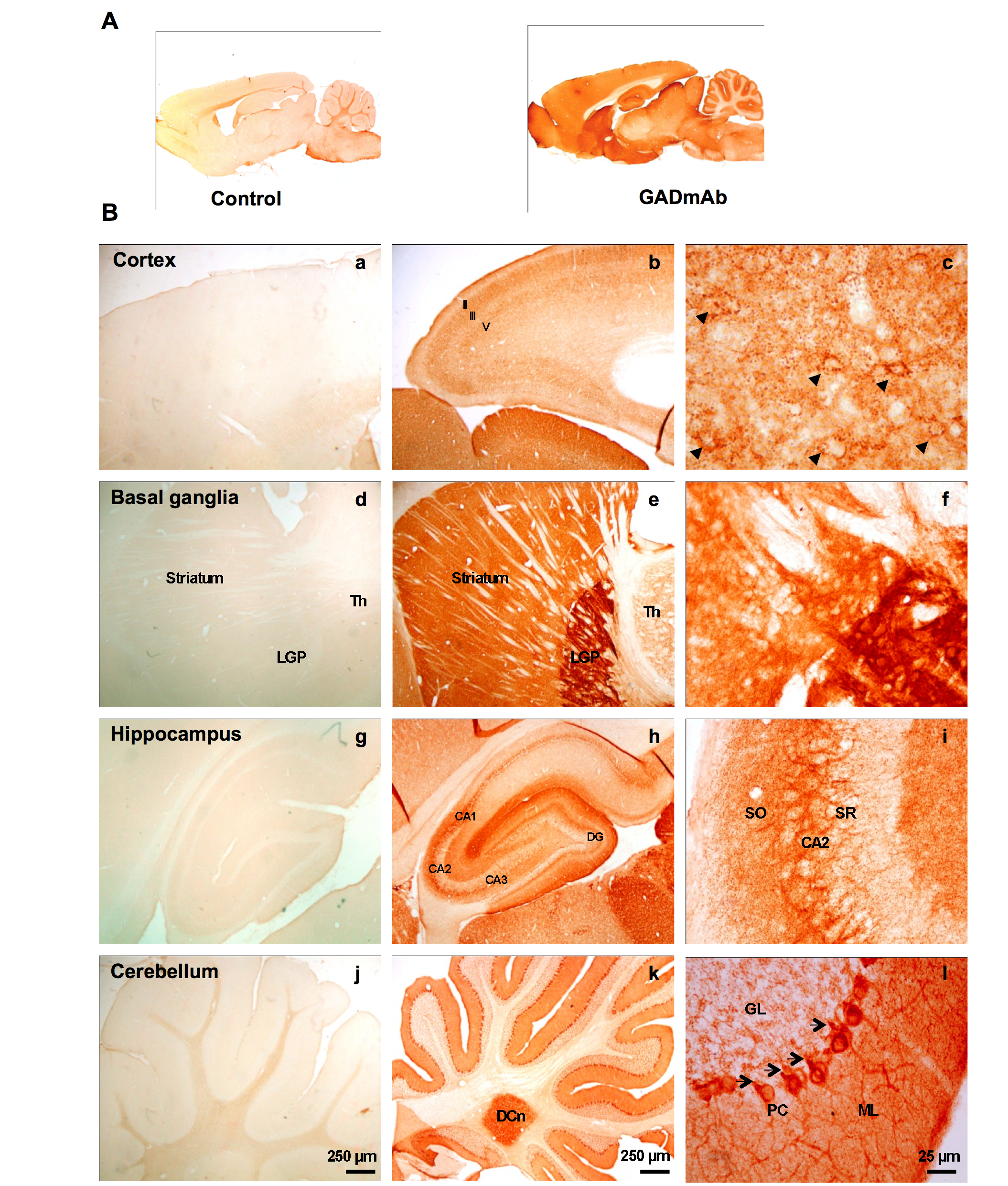

Supplement: Figure S2 — GAD immunoreactivity in rat brain sections. (A) Whole rat brain saggital sections stained with healthy control and GAD monoclonal antibodies (×10). Note the contrast in immunoreactivity. (B) Examples of immunoreactivity in saggital sections through cortex, basal ganglia, hippocampus and cerebellum. First column represents normal healthy serum. Second and third columns represent GADmAb. Note (c) immunoreactive dots outlining pyramidal cells in the cortex (arrow heads); (e) intense immunoreactivity of lateral globus pallidus (LGP) relative to the striatum and thalamus (Th) in basal ganglia; (h, i) staining surrounding pyramidal cells in the hippocampal regions CA1-3; (l) the dense accumulation of immunoreactivity at the axon hillock (small arrows) and puncta that outline the perikaryon and dendritic tree of Purkinje cells (PC), punctate staining in the molecular (ML) and granular layer, and (k) intense immunoreactivity in deep cerebellar nuclei (DCn). SO: stratum oriens; SR: stratum radiatum; DG: dentate gyrus. (TIF) [file pone.0072921.s002.tif]
